# Dr. Hall’s Remedies

**Published:** 1879-10

**Authors:** 


### VALUABLE REMEDIES.

***Constipation*** is a very common complaint, and it is the beginning of many fatal diseases of the vital organs, which are brought on by absorption into the blood of fecal matter which has been too long retained in the bowels.

***Piles, Fistula, and Cancer of the Rectum*** are also caused by CONSTIPATION, owing to the pressure of impacted feces in the lower portion of the rectum, which interrupts the circulation of the blood, and also to the excoriating nature of this retained matter, which acts like a strong acid on the delicate tissues. In the year 1856, the greatest surgeon in Europe, after years of careful investigation into the causes of Constipation and its remedy, introduced "NELATON'S SUPPOSITORY." It was hardly known in this country until ten years later.

***Nelaton's Suppository*** is a local application, which gives prompt and PERMANENT RELIEF in cases of CONSTIPATION or PILES, and causes no unpleasant disturbance in the system. Price 50 cents a box.

### *Dr. Hall's Complete Cure for Fever and Ague.*

The seat of this distressing complaint is in the LIVER. When the proper remedies are employed, there is no difficulty in curing the disease in from two to six days, so that it will not return, unless from the most careless indifference to the rules of health.

DR. HALL'S COMPLETE CURE FOR FEVER AND AGUE is warranted to contain neither quinine, arsenic, or other deleterious drugs. Price 50 cents a box.

### *Van Deren's Remedy for Diseases of Children.*

For Cholera Infantum and all Bowel troubles of children, this remedy has taken the lead of all other preparations, and no case of failure to do all it is recommended to do has ever been reported. It is, however, not sufficiently known that it is the most valuable of all remedies for children suffering from the many diseases that depend on a torpid condition of the Liver, and to which young children are liable at all seasons. Price 50 cents a box.

### *Dr. Hall's Old Time Liver Pills.*

These Pills cause no pain or uneasiness, and may be taken at any time without interruption to business or pleasure.

Their effect is to induce healthy action of the Liver, and to cleanse the stomach from those impure matters which induce disease. Price 25 cents a box.

### Dr. Hall's Troches for Coughs, Colds, Hoarseness, and Sore Throat.

The Cheapest and Best Troches in the market. Only 25 cents a box.

### *Dr. Hall's Snuff for Influenza and Catarrh.*

Taken frequently at the commencement of Influenza or Cold in the Head, it seldom fails to effect a cure in a few hours. It affords great relief in Chronic Catarrh. Price 25 cents a box.

Any of these remedies will be mailed free on receipt of price.

### *Dr. Hall's Spirometer.*

The best, cheapest, and most compact Spirometer in the market. The use of the Spirometer is the best means of exercising and strengthening the lungs, so that they may throw off disease or resist its attacks. Price $5.50.

### *Retarded Menstruation.*

HOMEWOOD, SCOTT CO., MISS., *Nov.* 12, 1872.

DR. J. R. STAFFORD: Enclosed you will find $2.50 for three more packages of your Iron and Sulphur Powders. One package of the last I sent for has made regular a young woman 22 years old, who had never menstruated but once before. Another package was used by my daughter, who was threatened with loss of sight from sore eyes. One package of the powders cured her. MRS. REBECCA WRIGHT.

Sold by Druggists. 1 package, 12 powders, $1.00; 6 packages, 72 powders, $5.00

Mailed free. **HALL & RUCKEL,**

218 GREENWICH ST., NEW YORK.

